# Predicting extubation success: still a conundrum?

**DOI:** 10.1038/s41390-022-02220-7

**Published:** 2022-08-19

**Authors:** Kristin N. Ferguson, David G. Tingay

**Affiliations:** 1 Neonatal Research, Murdoch Children’s Research Institute, Parkville, VIC Australia; 2 Department of Neonatology, The Royal Children’s Hospital, Parkville, VIC Australia; 3 Department of Paediatrics, The University of Melbourne, Parkville, VIC Australia

Mechanical ventilation via an endotracheal tube (ETT) is now largely reserved as a life-saving measure for premature infants not responding to non-invasive modes.^1^ Prolonged mechanical ventilation is associated with an increased risk of short- and long-term complications, including adverse neurodevelopmental outcomes, bronchopulmonary dysplasia and death.^2,3^ Unfortunately, extubation failure also confers significant respiratory and neurological risks and increases mortality.^4^ This often creates a clinical conundrum, made worse by the awareness that extubation failure occurs in a third of intubated preterm infants.^5^ This is in part because reliably determining extubation readiness at the bedside is difficult, and existing tests of extubation readiness are far from perfect.^6–8^ The Spontaneous Breathing Trial (SBT) has been widely adopted by clinicians for its ease of implementation at the bedside.^6,7,9^ The SBT involves determining whether an infant can maintain adequate oxygenation during a brief period of spontaneous breathing via the ETT during continuous positive end-expiratory pressure (CPAP).^7^ The rationale being that the imposed work of breathing during ETT CPAP is likely to be greater than that after extubation. Despite a high sensitivity (95%), the moderate specificity (62%) emphasises the limitations of the SBT to predict an infant who will fail extubation.^9^ Thus, clinicians must still rely on a degree of subjective clinical acumen, combining tests like the SBT with readily available clinical parameters and personal experience, to guide decisions on extubation readiness.

In this issue of *Pediatric Research* Williams et al. add further insight into the perennial dilemma facing clinicians caring for preterm infants: is now the right time to extubate?^10^ Williams et al. studied 48 infants born less than 37 weeks receiving invasive mechanical ventilation. When clinically deemed ready for extubation, a 10-min SBT was performed using ETT CPAP.^10^ Infants who passed the SBT were subsequently extubated and followed for 48 h (the study’s definition of extubation success). The novel aspect of the study was the inclusion of a quantifiable assessment of diaphragmatic activity using transcutaneous electromyography (EMG) 10 min before and during the SBT. The primary aim was to determine whether measuring diaphragmatic EMG during an SBT may be useful in predicting extubation failure. The authors found that diaphragmatic activity increased during the SBT and was different between those infants who succeeded extubation and those who did not.

Extubation is an active and intentional clinician decision in which the infant is given complete control over their own respiratory drive and effort. In doing so the clinician must determine whether a preterm infant has a patent airway, sufficient functional residual capacity and lung parenchyma to facilitate gas exchange, as well as the developmental readiness of thoracic muscles and central drive to support minute ventilation and end-expiratory volumes. As such, it is likely that a composite extubation readiness test, incorporating a series of physiological measures, will more accurately identify those at risk of extubation failure than the SBT alone.^6,8^ As the primary muscle of respiration, the diaphragm is a logical focus in preterm infants. Inadequate diaphragmatic activity will also influence many of the other barriers to post-extubation breathing, such as functional residual capacity and minute ventilation. Conversely, inadequate lung volumes and airway obstruction will increase the work of breathing and diaphragmatic activity. The emergence of transcutaneous EMG allows non-invasive assessment without the need for an oesophageal probe.^11,12^ The authors identified a 50% (1.2 μV) increase in EMG amplitude (a measure of diaphragmatic contraction) during the SBT. This is not surprising. It would be expected that infants will increase diaphragmatic activity during the imposed work of breathing of ETT CPAP generally. The challenge for this technology will be whether amplitude changes of 1–3 μV can be used by clinicians to differentiate an appropriate increase in respiratory effort versus excessive work of breathing related to atelectasis or airway insufficiency, especially when a degree of signal variability due to probe location or artefact in an active infant is to be expected.^13^

Three infants failed the SBT, and of the 45 who passed, a total of 32 remained extubated 48 h later (73% success rate). Overall developmental status of the lung was the most important predictor of extubation success, with success being greater as postmenstrual age increased.^6^ The inclusion of EMG appeared to be more important in infants <29 weeks. The area under the curve of the EMG signal (EMG_AUC_) was greater during the SBT in the 13 infants who failed extubation (11 infants <29 weeks). EMG_AUC_ is an indicator of the total amount of muscle activity, with greater values suggesting increased tonic diaphragmatic activity. Again, it is reassuring for the technology that extubation failure was associated with an increased EMG_AUC_ in the more immature infant. Infants <29 weeks’ gestation have a greater reliance on the diaphragm to support respiration, as well as a lower functional residual capacity, and a greater likelihood of parenchymal lung disease, impaired gas exchange and a patent ductus arteriosus. All of these are likely to impose greater stressors on the diaphragm.

Conclusions around the importance of increased EMG_AUC_ in infants <29 weeks’ gestation should be made with caution. Like other studies investigating transcutaneous EMG during extubation and non-invasive ventilation in preterm infants,^11,12^ the study of Williams et al. was not designed or powered to determine the ability of EMG to predict extubation failure (or success). The sample size being based upon an absolute change in electrical diaphragmatic activity, and not the clinical implications of that change. Furthermore, the EMG_AUC_ changes cannot be assumed to predict lung volume loss. Hopefully, future studies will focus on linking the diaphragmatic EMG activity to concomitant quantifiable measures of lung volume and mechanics such as electrical impedance tomography, tidal volume and required inflating pressure.^14,15^ This will be an important step towards not just understanding how the diaphragm responds to the challenges of extubation, but also developing a suite of physiological measures that might refine the precision of extubation readiness tests. The ultimate goal is to develop an extubation readiness bundle that can be evaluated in a properly powered interventional study.

Predicting readiness for extubation is just half of the problem. Understanding whether an infant is managing once extubated is equally important.^5^ EMG data beyond the immediate extubation period would have been useful and may ameliorate the impact of the low specificity (58%) in predicting failure. If these results can be replicated in larger studies, 42% of infants who were not extubated based upon EMG would have succeeded. It is possible that EMG and other measures of lung function may provide clinicians with the confidence to extubate if they could then use these same tools to guide changes in non-invasive ventilation management or modality. Ongoing EMG data would also aid in understanding the role of apnoea, which was not a prominent feature in this study, but is often attributed to extubation failure.

Avoiding unnecessary invasive ventilation remains one of the largest practical challenges in neonatal respiratory care, and is defined by clinical art as much as science. The study of Williams et al. demonstrates that measuring the work of breathing when being considered for extubation is feasible. This is an important step towards providing clinicians with a physiologically quantifiable dimension to extubation assessment. Appropriately powered studies designed to determine the efficacy and safety of diaphragmatic EMG to determine extubation choices will be needed before it can be advocated for clinical use.
